# ﻿A revision of *Chrysso* O. Pickard-Cambridge, 1882 *sensu lato* (Araneae, Theridiidae)

**DOI:** 10.3897/zookeys.1266.172838

**Published:** 2026-01-09

**Authors:** Changhao Hu, Rui Zhong, Jie Liu, Zichang Li

**Affiliations:** 1 Arachnid Resource Centre of Hubei Province & Hubei Key Laboratory of Regional Development and Environmental Response, Faculty of Resources and Environmental Science, Hubei University, Wuhan 430062, China Hubei University Wuhan China; 2 Centre for Behavioural Ecology and Evolution, School of Life Sciences, Hubei University, Wuhan 430062, China Hubei University Wuhan China

**Keywords:** Biodiversity, morphology, new genus, new species, revalidation, taxonomy

## Abstract

A revision of *Chrysso* O. Pickard-Cambridge, 1882 *sensu lato* is presented based on specimens collected from China. A new genus, *Megama***gen. nov.**, is established, based on *Meg. decimaculata***sp. nov.** (male, female) from Hubei, China as the type species, with an additional species *Meg. bimaculata* (Yoshida, 1998), **comb. nov.** Two new species of *Meotipa* Simon, 1895, *Meo. cordata***sp. nov.** and *Meo. vittiforma***sp. nov.**, are described, and four new combinations are proposed. The genus *Physcoa* Thorell, 1895, stat. rev. is revalidated, with its type species *P. scintillans* Thorell, 1895, comb. rev., along with seven new combinations. All the new combinations proposed in the current paper are transferred from the genus *Chrysso*. Diagnoses for the four genera are provided, along with diagnoses, descriptions, and photos of the new species.

## ﻿Introduction

The genus *Chrysso* O. Pickard-Cambridge, 1882, established with *Chr. albomaculata* O. Pickard-Cambridge, 1882 as the type species, is one of the most diverse genera in the family Theridiidae Sundevall, 1833, comprising 61 extant species distributed in Asia, Europe, and the Americas (WSC 2025). Members of *Chrysso* are characterized by a posterior dorsal extension of the opisthosoma, and mainly inhabit the underside of leaves, where they build a star-shaped web (Zhu 1998; Agnarsson 2004).

Levi and Levi (1962) considered *Physcoa* Thorell, 1895 as a junior synonym of *Chrysso*. Yoshida (2001) accepted this presumption after examining the type of *Argyria venusta* Yaginuma, 1957 (*Chrysso venusta*) and considered it a junior synonym of *Chrysso scintillans* (Thorell, 1895), the type species of *Physcoa*. Both Simon (1895) and Levi and Levi (1962) considered *Chrysso* a senior synonym of *Meotipa*Simon 1895, but this view was rejected by Deeleman-Reinhold (2009). In addition, Yoshida (2009) established the genus *Chikunia* Yoshida, 2009 based on species previously belonging to the genus *Chrysso* (Grinsted et al. 2012; Kumada and Ono 2023). Although some early studies partially resolved the taxonomic problem of *Chrysso*, many species currently placed in this genus do not actually belong to it, and a revision of the genus is needed (Levi 1957, 1962; Zhang and Zhang 2012; Vanuytven 2021).

While examining theridiid specimens from China, a new species resembling *Chr. bimaculata* Yoshida, 1998 was identified. These two species differ markedly from *Chr. albomaculata*, and therefore we establish a new genus to accommodate them. In addition, two new species belonging to *Meotipa* were identified, and *Chr. lingchuanensis* Zhu & Zhang, 1992 together with three *Chrysso* from Philippines are transferred to *Meotipa* based on the presence of spines on the opisthosoma. Furthermore, some species currently placed in *Chrysso*, such as *Chr. scintillans*, exhibit morphological characters that are clearly different from those of *Chr. albomaculata*. Accordingly, we revalidate the genus *Physcoa* and transfer seven *Chrysso* species that are morphologically similar to it.

## ﻿Material and methods

The specimens examined in this study are deposited in the Centre for Behavioral Ecology and Evolution (CBEE), School of Life Sciences, Hubei University, Wuhan, China. Specimens were examined using Olympus SZX7 stereomicroscopes. Photographs were taken with an Olympus BX51 microscope. Male palps were examined and photographed after dissection and expansion in warmed lactic acid. Epigynes were dissected from the spiders’ bodies and treated with a warmed 0.1 mg/ml Protease K solution before studying. Membranous structures of epigynes were stained with Amido black 10B. All morphological measurements were taken using a Leica M205 C stereomicroscope. Eye diameters were measured at the widest point. Leg measurements are given as total length (femur, patella, tibia, metatarsus, tarsus). All measurements are given in millimetres (mm). The terminologies used in text and figure legends follow Agnarsson et al. (2007).

Abbreviations:

**A** = atrium;

**ALE** = anterior lateral eye;

**AME** = anterior median eye;

**C** = conductor;

**CD** = copulatory duct;

**Ch** = cymbial hood;

**CO** = copulatory opening;

**CP** = cymbial projection;

**E** = embolus;

**EBH** = embolic basal hook;

**EP** = embolic projection;

**FD** = fertilization duct;

**MA** = median apophysis;

**PLE** = posterior lateral eye;

**PME** = posterior median eye;

**S** = spermatheca;

**ST** = subtegulum;

**T** = tegulum;

**TA** = tegular apophysis;

**Tb** = trichobothria;

**TO** = tarsal organ;

**TP** = tegular pit;

**I**, **II**, **III**, **IV** = legs I–IV.

## ﻿Results

### ﻿Taxonomy

**Family Theridiidae Sundevall, 1833**

#### ﻿Genus Chrysso

Taxon classificationAnimaliaAraneaeTheridiidae

O. Pickard-Cambridge, 1882

0BECD2CA-07F3-5F2B-97AA-F096614D651F

Chrysso : O. Pickard-Cambridge, 1882: 429.
Arctachaea : Levi, 1957: 102 (synonym by Levi and Levi 1962: 16).
Argyria : Yaginuma, 1957: 11 (synonym by Levi and Levi 1962: 16).
Argyroaster : Yaginuma, 1958: 37 (synonym by Levi and Levi 1962: 16).

##### Type species.

*Chrysso albomaculata* O. Pickard-Cambridge, 1882 from the Americas.

##### Remarks.

The morphology of the copulatory organs in “*Chrysso*” species is highly variable. Therefore, we choose the type species, *Chr. albomaculata*, along with eight morphologically similar species from North and South America as the true *Chrysso*: *Chr. albomaculata* O. Pickard-Cambridge, 1882, *Chr. diplosticha* Chamberlin & Ivie, 1936, *Chr. huanuco* Levi, 1957, *Chr. indicifera* Chamberlin & Ivie, 1936, *Chr. mariae* Levi, 1957, *Chr. sulcata* (Keyserling, 1884), *Chr. vallensis* Levi, 1957, and *Chr. vexabilis* Keyserling, 1884.

##### Diagnosis.

We provide the diagnostic characteristics of the true *Chrysso* in the current paper. The true *Chrysso* (figs 1–7, 9, 11, 13, 15, 21–31, 34, 35 in Levi 1957; figs 7, 8, 12 in Buckup and Marques 1996) can be distinguished from other theridiid genera by the following combination of characteristics: (1) opisthosoma longer than wide or high, posterior part extending beyond spinnerets; (2) median apophysis almost as large as tegular apophysis; (3) embolus arising from prolateral part of embolic base and retrolaterally elongating, then curved in clockwise direction; (4) copulatory ducts sac-like; and (5) spermathecae spherical.

#### ﻿Genus Megama gen. nov.

Taxon classificationAnimaliaAraneaeTheridiidae

07CF519C-4AD1-5030-B553-3E2A3CED2834

https://zoobank.org/82B6D88D-7A6B-43FA-8E98-47238318928C

##### Type species.

*Megama decimaculata* sp. nov. from China.

##### Etymology.

The generic name is a combination of the Greek word *mega* (large) and *ma* (abbreviation of median apophysis), referring to the large median apophysis of this new genus. The gender is feminine.

##### Diagnosis.

Members of the genus *Megama* gen. nov. can be distinguished from the true *Chrysso* (cf. Figs 1–3 and figs 1–7, 9, 11, 13, 15, 21–31, 34, 35 in Levi 1957; figs 7, 8, 12 in Buckup and Marques 1996) by: (1) median apophysis posteriorly elongating, with a thin and long dorsal part almost as long as ventral part (vs median apophysis triangular, without elongation); (2) tegular apophysis tiny, almost 1/5–1/3 of the median apophysis (vs almost as large as median apophysis); (3) embolus relatively shorter than bulb, with a pointed embolic projection (vs relatively longer than bulb, lacking embolic projection); (4) copulatory ducts tube-like (vs sac-like); and (5) spermathecae longer than wide or wider than long (vs spherical).

**Figure 1.**
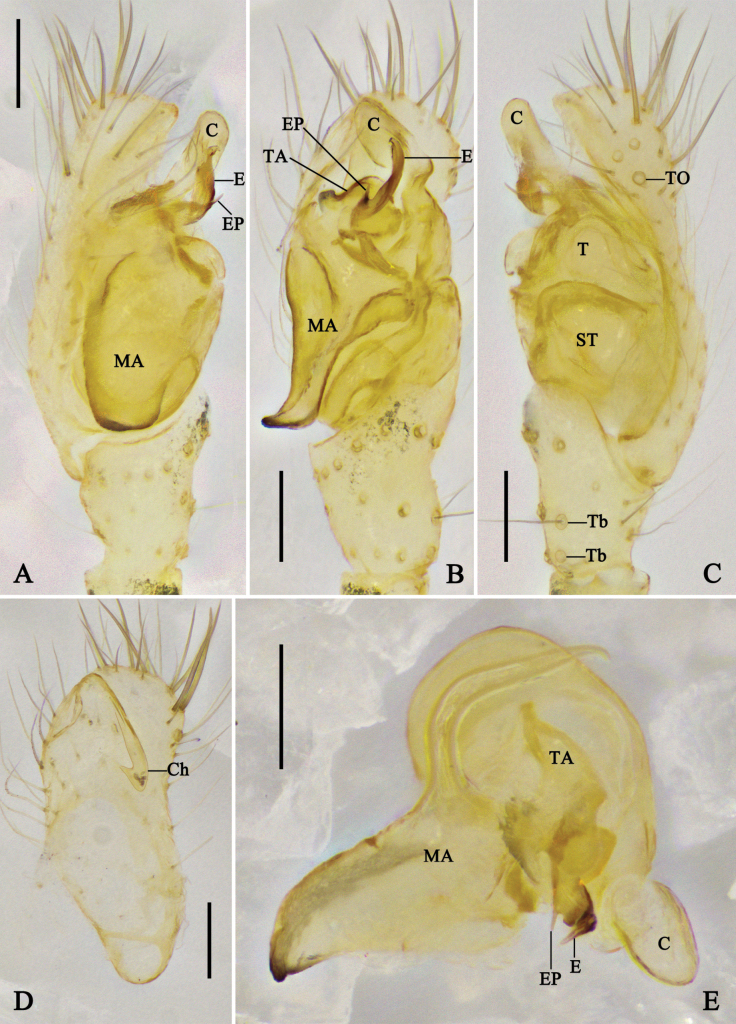
Left male palp of *Megama decimaculata* sp. nov. **A.** Prolateral view; **B.** Ventral view; **C.** Retrolateral view; **D.** Cymbium, ventral view; **E.** Expanded bulb, apical view. Abbreviations: C = conductor; Ch = cymbial hood; E = embolus; EP = embolic projection; MA = median apophysis; ST = subtegulum; T = tegulum; TA = tegular apophysis; Tb = trichobothria; TO = tarsal organ. Scale bars: 0.1 mm.

**Figure 2.**
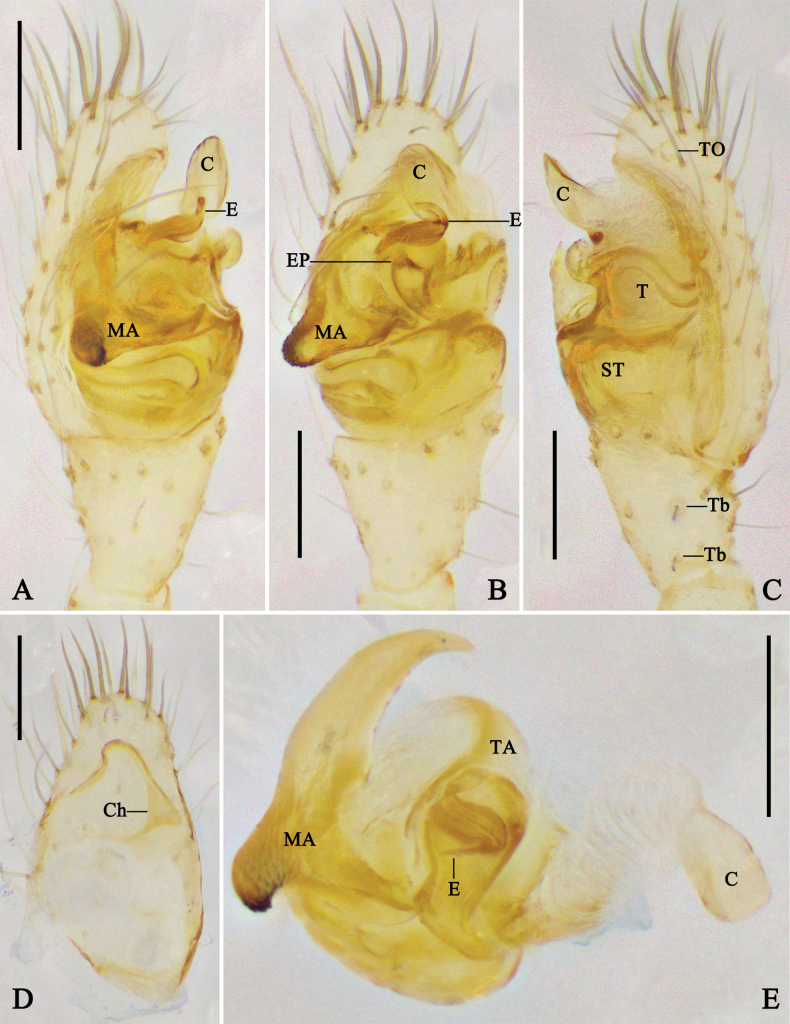
Left male palp of *Megama bimaculata* (Yoshida, 1998), comb. nov. **A.** Prolateral view; **B.** Ventral view; **C.** Retrolateral view; **D.** Cymbium, ventral view; **E.** Expanded bulb, apical view. Abbreviations: C = conductor; Ch = cymbial hood; E = embolus; EP = embolic projection; MA = median apophysis; ST = subtegulum; T = tegulum; TA = tegular apophysis; Tb = trichobothria; TO = tarsal organ. Scale bars: 0.1 mm.

**Figure 3.**
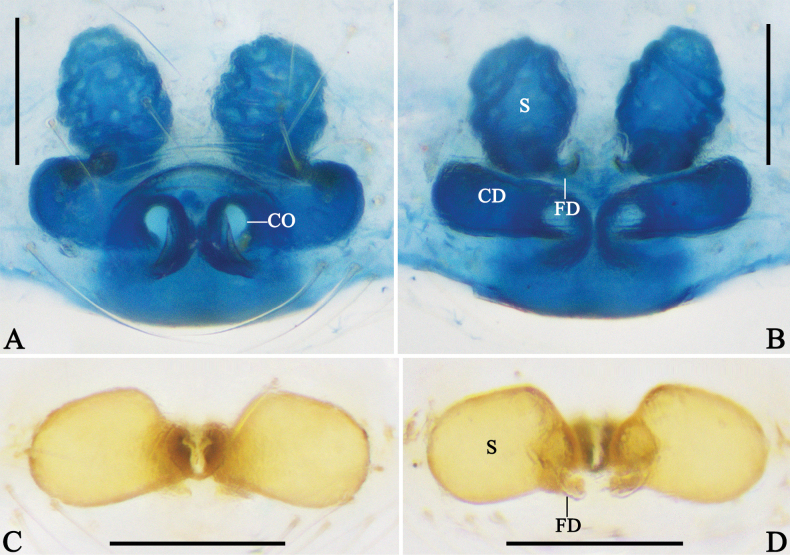
Female genitalia of *Megama* spp. **A, B.***Meg. decimaculata* sp. nov.; **C, D.***Meg. bimaculata* (Yoshida, 1998), comb. nov.; **A, C.** Epigyne, ventral view; **B, D.** Vulva, dorsal view. Abbreviations: CO = copulatory opening; CD = copulatory duct; FD = fertilization duct; S = spermatheca. Scale bars: 0.1 mm.

##### Description.

**Figure 4.**
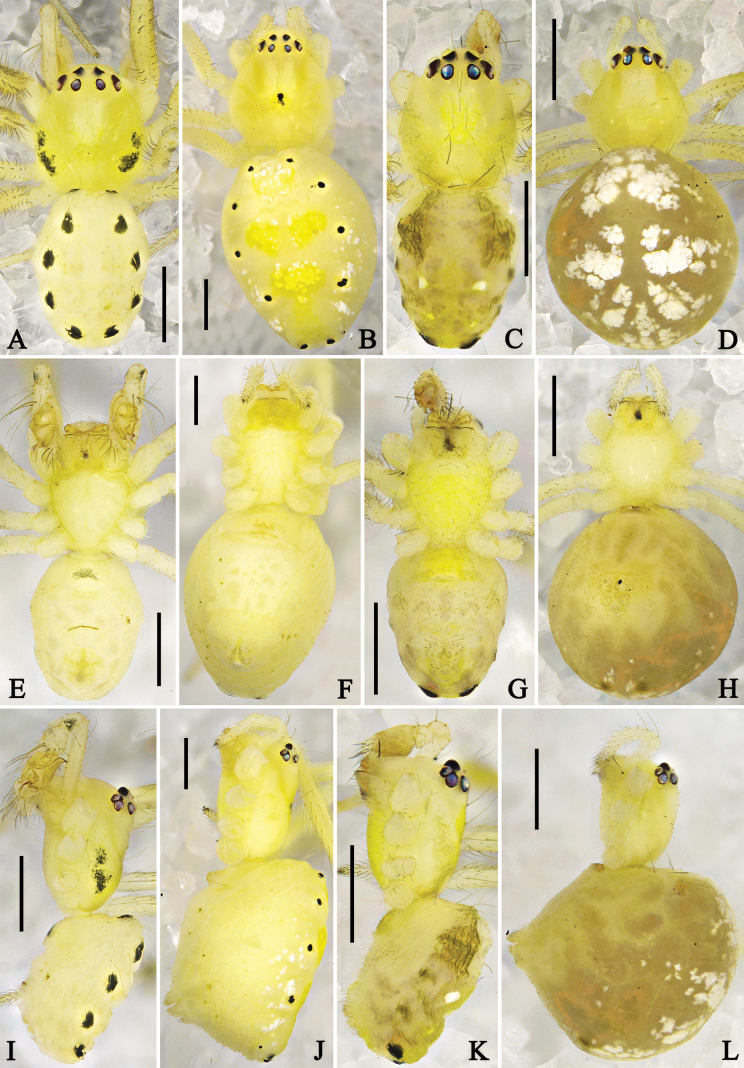
Habitus of *Megama* spp. **A, E, I.** Male of *Meg. decimaculata* sp. nov.; **B, F, J.** Female of *Meg. decimaculata* sp. nov.; **C, G, K.** Male of *Meg. bimaculata* (Yoshida, 1998), comb. nov.; **D, H, L.** Female of *Meg. bimaculata* (Yoshida, 1998), comb. nov.; **A–D.** Dorsal view; **E–H.** Ventral view; **I–L.** Lateral view. Scale bars: 0.5 mm.

***Habitus*** (Fig. 4): male total length 1.48–1.88, female 1.80–3.20. Habitus generally light yellow. Carapace oval. Sternum triangular. Chelicerae with two promarginal teeth, without retromarginal teeth. Leg formula 1423. Retrolateral part of patella of legs with apophysis. Opisthosoma oval, posterior part extending beyond spinnerets; dorsal part of opisthosoma with black spots. Colulus or colular setae absent.

***Male palp*** (Figs 1, 2): tibia almost half the length of cymbium, basal retrolateral part with two trichobothria. Cymbium longer than wide, with the basal part narrowest; cymbial hood longer than 1/4 length of cymbium, with curved apical part; tarsal organ larger than setal sockets. Subtegulum and tegulum with prolateral part shorter than retrolateral part. Median apophysis with triangular ventral part, and long dorsal part almost as long as ventral part. Tegular apophysis tiny, almost 1/5–1/3 of the median apophysis. Conductor cow-ear-like, connecting with tegulum via membrane. Embolus with a pointed projection; embolic tip curved in anticlockwise direction.

***Epigyne*** (Fig. 3): epigyne slightly sclerotized. Copulatory ducts tube-like, thinner than spermathecae. Spermathecae oval. Fertilization ducts curved, arising from posterior part of spermathecae.

##### Composition.

*Megama decimaculata* sp. nov. and *Meg. bimaculata* (Yoshida, 1998), comb. nov.

##### Distribution.

China, Japan.

#### ﻿ Megama decimaculata sp. nov.

Taxon classificationAnimaliaAraneaeTheridiidae

03F29F4B-AD23-504A-8987-313BE9D8AD8A

https://zoobank.org/1490F466-5D1D-4CFE-B24A-FC30654FFA97

Figs 1, 3A, B, 4A, B, E, F, I, J

##### Type material.

***Holotype*** male (QZMS05450): China – Hubei Province • Enshi Tujia and Miao Autonomous Prefecture, Xuan’en County, Qizimeishan National Nature Reserve, Chunmuying Town, Huoshaobao; 30.0242°N, 109.7565°E; elev. 1919 m; 1 June 2024; C.H. Hu and M. Wei leg. ***Paratypes*** 1 male, 6 females (QZMS05451–05457): same data as for holotype.

##### Etymology.

The specific name is a combination of the Latin prefix *deci*- (ten) and the Latin adjective *maculatus* (with spots), referring to the 10 black spots on the dorsal opisthosoma.

##### Diagnosis.

*Megama decimaculata* sp. nov. can be distinguished from *Meg. bimaculata* (Yoshida, 1998), comb. nov. (cf. Figs 1, 3A, B and Figs 2, 3C, D) by: (1) median apophysis almost 2/3 the length of bulb, prolateral angle of median apophysis pointed (vs median apophysis almost 1/3 the length of bulb, prolateral angle of median apophysis rounded with rough surface); (2) embolic tip pointing to 12 o’clock direction in ventral view (vs 10 o’clock direction); (3) copulatory ducts long and obvious (vs very short, unobvious); and (4) spermathecae longer than wide (vs wider than long).

##### Description.

**Male**: total length 1.88; carapace length 0.86, width 0.74; opisthosoma length 1.00, width 0.77; eye diameters: AME 0.06, ALE 0.06, PME 0.07, PLE 0.07; eye interdistances: AME–AME 0.09, ALE–AME 0.04, PME–PME 0.10, PLE–PME 0.07, AME–PME 0.06, ALE–PLE 0.01; leg measurements: I 5.98 (1.70, 0.31, 1.53, 1.84, 0.60), II 3.53 (1.06, 0.25, 0.81, 0.95, 0.46), III 2.31 (0.72, 0.20, 0.46, 0.60, 0.33), IV 3.56 (1.09, 0.22, 0.87, 0.96, 0.42).

***Palp*** (Fig. 1): median apophysis triangular, with long dorsal part almost as long as ventral part. Tegular apophysis tiny, almost 1/5 of the median apophysis. Embolic tip pointing to 12 o’clock direction. Other characters as in generic description.

***Colouration in ethanol*** (Fig. 4A, E, I): carapace laterally with black markings. Distal part of patella, tibia, and metatarsus of legs with black spots. Dorsal opisthosoma with five pairs black spots. Other characters as in generic description.

**Female**: total length 3.20; carapace length 1.14, width 1.05; opisthosoma length 1.97, width 1.52; eye diameters: AME 0.05, ALE 0.06, PME 0.06, PLE 0.06; eye interdistances: AME–AME 0.12, ALE–AME 0.05, PME–PME 0.10, PLE–PME 0.07, AME–PME 0.07, ALE–PLE 0.01; leg measurements: I 8.82 (2.63, 0.37, 2.25, 2.84, 0.73), II 4.91 (1.62, 0.26, 1.06, 1.40, 0.57), III 3.11 (0.96, 0.30, 0.60, 0.81, 0.44), IV 5.69 (1.80, 0.34, 1.35, 1.62, 0.58).

***Epigyne*** (Fig. 3A, B): epigyne medially with septum. Copulatory ducts thick and long, almost as long as spermathecae. Spermathecae anteriorly located. Other characters as in generic description.

***Colouration in ethanol*** (Fig. 4B, F, J): as in male, but carapace generally light yellow, dorsal part of opisthosoma with white and yellow patches.

##### Distribution.

Known only from the type locality (China: Hubei).

#### ﻿ Megama bimaculata

Taxon classificationAnimaliaAraneaeTheridiidae

(Yoshida, 1998) comb. nov.

5058C35A-9F53-58F8-86C1-E445FB2CDA24

Figs 2, 3C, D, 4C, D, G, H, K, L

Chrysso bimaculata : Yoshida 1998: 105, figs 1–6 (male, female); Yoshida 2003: 125, figs 330–335 (male, female); Yoshida 2009: 378, figs 203, 204 (male, female); Zhang and Zhang 2012: 28, figs 13–17 (male, female).

##### Material examined.

5 males, 11 females (QZMS00947, 00981, 01024, 01392, 02586, 02736, 02938, 03189, 03564, 04599–04604): China – Hubei Province • Enshi Tujia and Miao Autonomous Prefecture, Xuan’en County, Qizimeishan National Nature Reserve; 29.6953°N–30.0292°N, 109.5919°E–109.7565°E; elev. 585–1919 m; 27 June–20 July 2023; C.H. Hu and M. Wei leg.

##### Diagnosis.

See above diagnosis under *Meg. decimaculata* sp. nov.

##### Description.

See Zhang and Zhang (2012).

##### Distribution.

China (Hubei, new provincial record; Hainan), Japan.

#### ﻿Genus Meotipa

Taxon classificationAnimaliaAraneaeTheridiidae

Simon, 1895

ED418323-33A3-5A1D-A0CE-72671E15289C

Meotipa : Simon 1895: 133.

##### Type species.

*Meotipa picturata* Simon, 1895 from India.

##### Diagnosis.

See Deeleman-Reinhold (2009).

##### New combination.

*Meotipa anei* (Barrion & Litsinger, 1995), comb. nov., *Meo. isumbo* (Barrion & Litsinger, 1995), comb. nov., *Meo. lingchuanensis* (Zhu & Zhang, 1992), comb. nov., and *Meo. tiboli* (Barrion & Litsinger, 1995), comb. nov.

##### Remarks.

The most conspicuous characteristic of the genus *Meotipa* is the presence of flattened spines on the opisthosoma and legs. Although the specimens of *Chrysso anei*, *Chr. isumbo*, and *Chr. tiboli* were unavailable for examination, the original illustrations and descriptions of these species (figs 250a, 251a, 252a in Barrion and Litsinger 1995) clearly show spines on the dorsal opisthosoma. Therefore, we transfer these three species to *Meotipa* herein.

##### Distribution.

East, South, and Southeast Asia; Pacific Islands (WSC 2025).

#### ﻿ Meotipa cordata sp. nov.

Taxon classificationAnimaliaAraneaeTheridiidae

290D1099-13BF-570C-A33A-2EFAD59A44CD

https://zoobank.org/E6BE91A7-17B7-42D8-AB67-6944D3863A3B

**Figure 5.**
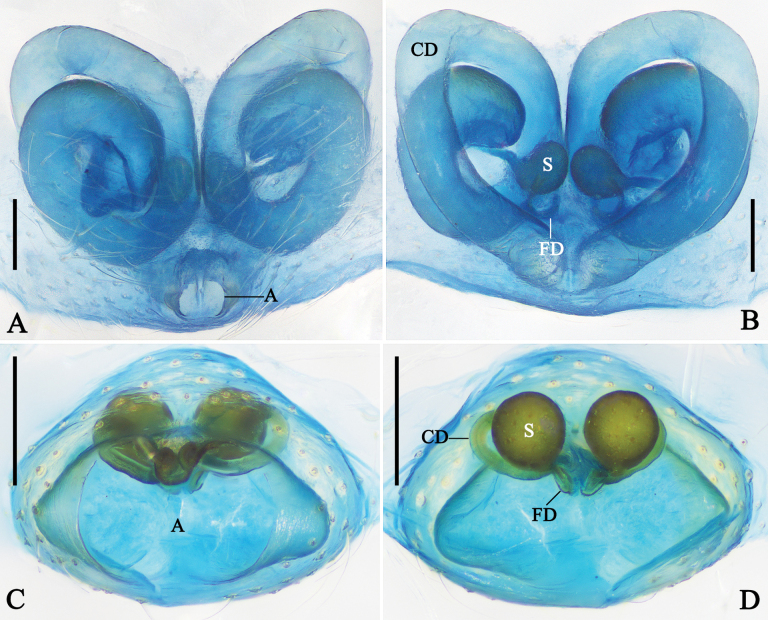
Female genitalia of *Meotipa* spp. **A, B.***Meo. cordata* sp. nov.; **C, D.***Meo. vittiforma* sp. nov.; **A, C.** Epigyne, ventral view; **B, D.** Vulva, dorsal view. Abbreviations: A = atrium; CD = copulatory duct; FD = fertilization duct; S = spermatheca. Scale bars: 0.1 mm.

Figs 5A, B, 7A, E, I

##### Type material.

**Holotype** female (QZMS01025): China – Hubei Province • Enshi Tujia and Miao Autonomous Prefecture, Xuan’en County, Qizimeishan National Nature Reserve, Changtanhe Dong Autonomous Town, Shanyangxi, 30.0764°N, 109.7454°E, elev. 810 m, 5 July 2023, C.H. Hu and M. Wei leg.

##### Etymology.

The specific name is derived from the Latin word *cordatus*, meaning “heart-shaped”, and refers the shape of the copulatory ducts.

##### Diagnosis.

The female of *Meo. cordata* sp. nov. can be distinguished from all congeners by: (1) the diameter of atrium, main part of copulatory ducts and spermathecae almost equal; and (2) copulatory ducts heart shaped.

##### Description.

**Female**: total length 3.19; carapace length 0.85, width 1.09; opisthosoma length 2.33, width 2.21; eye diameters: AME 0.07, ALE 0.09, PME 0.11, PLE 0.11; eye interdistances: AME–AME 0.10, ALE–AME 0.01, PME–PME 0.07, PLE–PME 0.03, AME–PME 0.05, ALE–PLE 0.00; leg measurements: I 7.52 (2.67, 0.35, 1.35, 2.51, 0.64), II 4.34 (1.45, 0.27, 0.79, 1.35, 0.48), III 2.88 (1.00, 0.24, 0.57, 0.66, 0.41), IV 5.33 (1.79, 0.34, 1.02, 1.70, 0.48). Leg formula 1423.

***Epigyne*** (Fig. 5A, B): epigyne posteriorly with a rounded atrium. Main part of copulatory ducts almost as wide as atrium, heart-shaped, coiled twice; distal part thin. Spermathecae kidney-shaped, medially situated, almost as wide as main part of copulatory ducts. Fertilization ducts arising from posterior part of spermathecae.

***Colouration in ethanol*** (Fig. 7A, E, I): carapace rounded and pale yellow, laterally with reddish-brown markings. Sternum, chelicerae, and labium pale yellow. Legs and palp pale yellow, with black, flattened spines (missing), black markings and reddish-brown spots. Opisthosoma rounded and pale yellow, with white patches and black spots; dorsum with black flattened spines (missing), laterally with two pairs humps and posteriorly with a black, tail-shaped extension.

**Male**: unknown.

##### Distribution.

Known only from the type locality (China: Hubei).

#### ﻿ Meotipa lingchuanensis

Taxon classificationAnimaliaAraneaeTheridiidae

(Zhu & Zhang, 1992) comb. nov.

F0BAE54B-D1A0-535F-936C-DC5FFB71EB68

**Figure 6.**
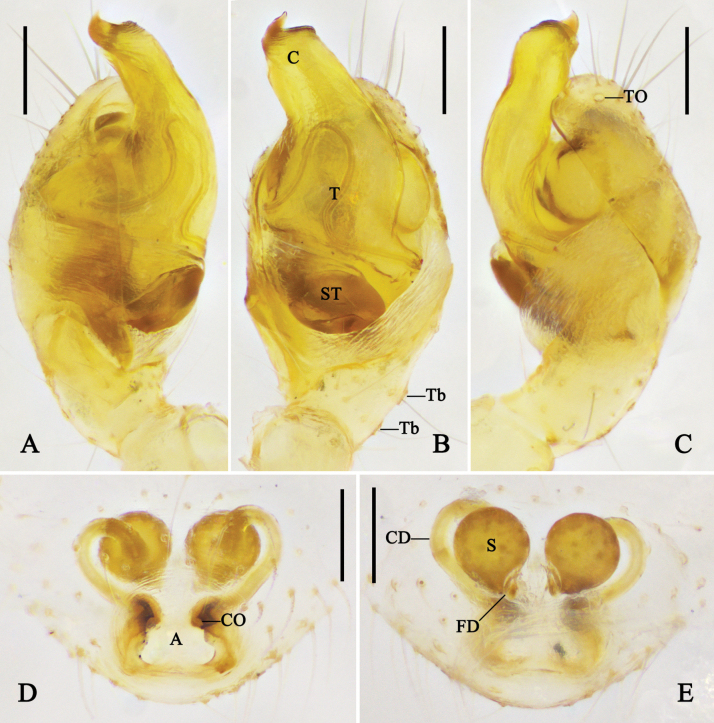
Copulatory organs of *Meotipa lingchuanensis* (Zhu & Zhang, 1992), comb. nov. **A–C.** Left male palp (**A.** Prolateral view; **B.** Ventral view; **C.** Retrolateral view); **D.** Epigyne, ventral view; **E.** Vulva, dorsal view. Abbreviations: C = conductor; CD = copulatory duct; FD = fertilization duct; S = spermatheca; ST = subtegulum; T = tegulum; Tb = trichobothria; TO = tarsal organ. Scale bars: 0.1 mm.

**Figure 7.**
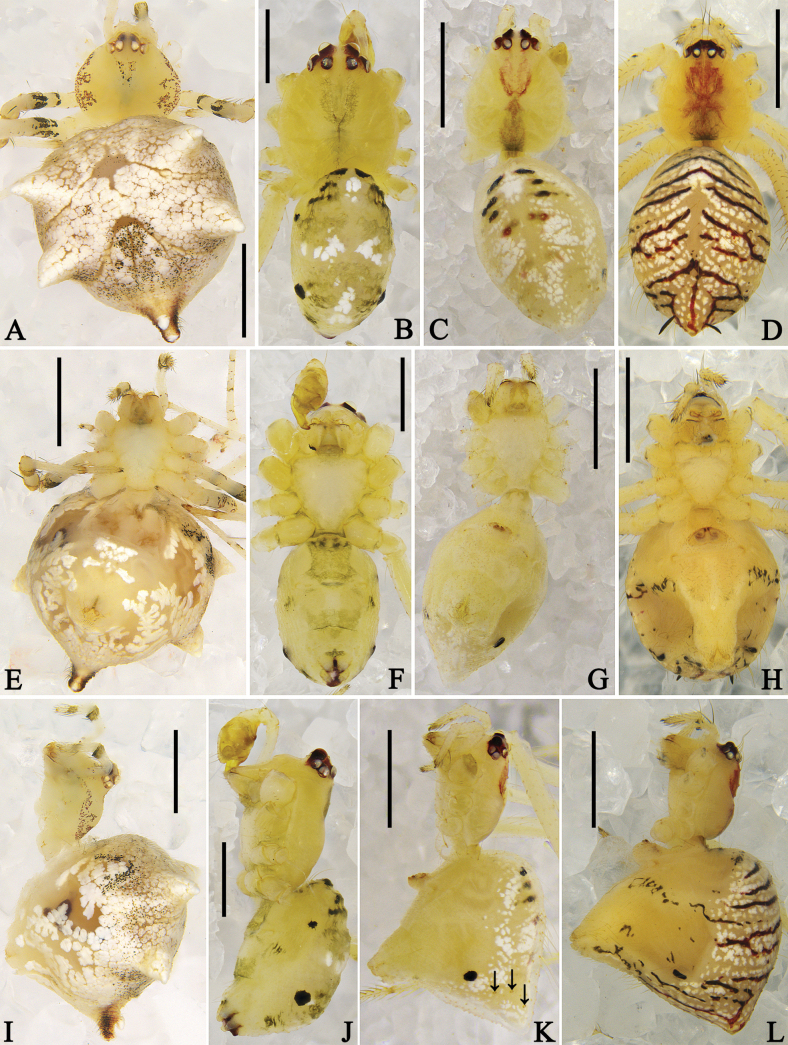
Habitus of *Meotipa* spp. **A, E, I.** Female of *Meo. cordata* sp. nov.; **B, F, J.** Male of *Meo. lingchuanensis* (Zhu & Zhang, 1992), comb. nov.; **C, G, K.** Female of *Meo. lingchuanensis* (Zhu & Zhang, 1992), comb. nov. (arrows in K point to the spine sockets); **D, H, L.** Female of *Meo. vittiforma* sp. nov.; **A–D.** Dorsal view; **E–H.** Ventral view; **I–L.** Lateral view. Scale bars: 1 mm (**A, C–E, G–I, K, L**); 0.5 mm (**B, F, J**).

Figs 6, 7B, C, F, G, J, K

Chrysso lingchuanensis : Zhu and Zhang 1992: 22, fig. 2A–D (female); Zhu 1998: 55, fig. 29A–F (male, female); Song et al. 1999: 103, fig. 49E, F, M, N (male, female); Lin et al. 2023: 521 (synonym of Chr. hyoshidai).
Chrysso hyoshidai : Barrion et al. 2013: 34, fig. 38A–E (female).

##### Material examined.

12 males, 19 females (PWFJ2024016–2024046): China – Fujian Province • Zhangzhou City, Zhangpu County, Futouwan; 23.9287°N, 117.6626°E; elev. 20 m; 14 July 2024; H.L. Chen et al. leg.

##### Diagnosis.

*Meotipa lingchuanensis* (Zhu & Zhang, 1992), comb. nov. is similar to *Meo. pseudomultuma* Liang, Yin & Xu, 2024 (cf. Fig. 6 and figs 3D–F, 4, 5D–F, 6 in Liang et al. 2024) in having a pointed conductor tip and laterally situated copulatory ducts, but it can be distinguished from *Meo. pseudomultuma* by: (1) sperm duct in tegulum strongly curved (vs slightly curved); (2) conductor tip curved in ventral view (vs straight); (3) conductor wide, almost 1/2 the width of bulb (vs narrow, almost 1/4 the width of bulb); (4) epigynal atrium present (vs absent); and (5) posterior margin of epigyne curved (vs straight).

##### Description.

See Zhu (1998).

##### Remarks.

**Figure 8.**
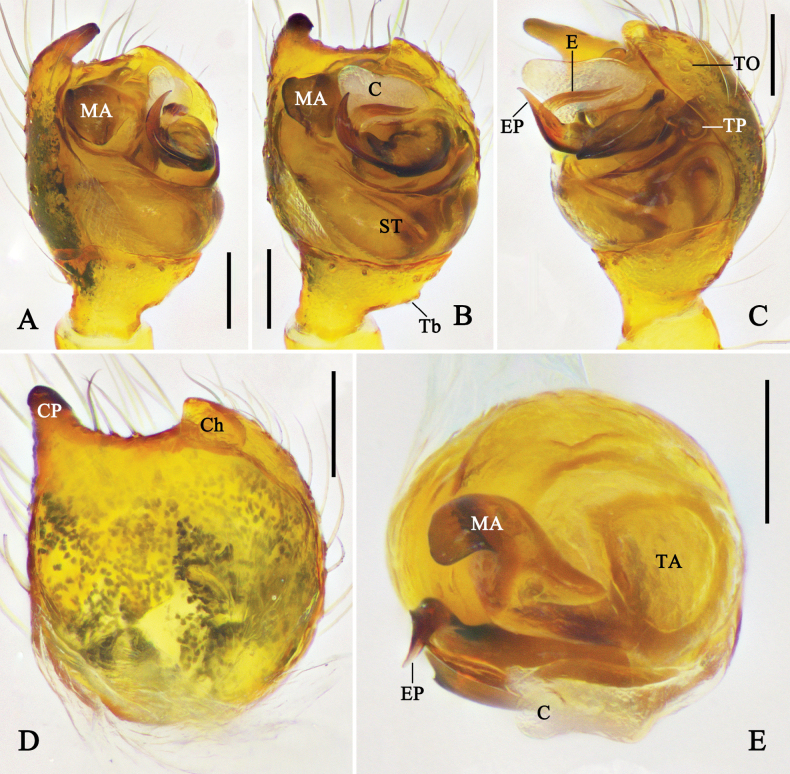
Left male palp of *Physcoa oxycera* (Zhu & Song, 1993), comb. nov. **A.** Prolateral view; **B.** Ventral view; **C.** Retrolateral view; **D.** Cymbium, ventral view; **E.** Expanded bulb, apical view. Abbreviations: C = conductor; Ch = cymbial hood; CP = cymbial projection; E = embolus; EP = embolic projection; MA = median apophysis; ST = subtegulum; T = tegulum; TA = tegular apophysis; Tb = trichobothria; TO = tarsal organ; TP = tegular pit. Scale bars: 0.1 mm.

This species possesses three pairs of spines on the posterior extension of the dorsal opisthosoma (arrows in Fig. 7K point to the spine sockets, but the spines are missing; fig. 29B in Zhu 1998), and the copulatory organs of this species are similar to the generotype (cf. Fig. 6 and figs 8G–I, 9C, D in Benjamin and Tharmarajan 2024): i.e. the embolus is surrounded by the conductor. Therefore, we transfer this species to the genus *Meotipa*.

##### Distribution.

China (Fujian, new provincial record; Guangdong; Guangxi; Hainan; Jiangxi).

#### ﻿ Meotipa vittiforma sp. nov.

Taxon classificationAnimaliaAraneaeTheridiidae

5C9127FE-CB29-5233-9356-E4205D70E2B2

https://zoobank.org/BC1A18D2-F754-446A-A730-C5A4E68E16E1

Figs 5C, D, 7D, H, L

##### Type material.

***Holotype*** female (LJ201800979): China – Hainan Province • Diaoluoshan; 18.7814°N, 109.5161°E, elev. 136 m, 1–31 March 2018, F.X. Liu and Z.C. Li leg.

##### Etymology.

The specific name is derived from the Latin word *vittiforma*, meaning “banded” and refers the reddish-brown transverse lines on the dorsal opisthosoma.

##### Diagnosis.

The female of *Meotipa vittiforma* sp. nov. is similar to *Meo. pseudomultuma* Liang, Yin & Xu, 2024 (cf. Figs 5C, D and figs 3D–F, 4 in Liang et al. 2024) in having similar copulatory duct surrounding spherical spermatheca, but it can be distinguished from *Meo. pseudomultuma* by: (1) epigyne with an atrium (vs without atrium); and (2) copulatory openings touching (vs separated).

##### Description.

**Female**: total length 2.86; carapace length 1.07, width 0.92; opisthosoma length 1.81, width 1.56; eye diameters: AME 0.08, ALE 0.06, PME 0.08, PLE 0.07; eye interdistances: AME–AME 0.08, ALE–AME 0.02, PME–PME 0.06, PLE–PME 0.08, AME–PME 0.07, ALE–PLE 0.01; leg measurements: I 7.31 (2.63, 0.32, 1.34, 2.40, 0.62), II 4.14 (1.44, 0.26, 0.65, 1.33, 0.46), III 2.70 (0.98, 0.23, 0.48, 0.63, 0.38), IV 5.12 (1.75, 0.33, 0.99, 1.58, 0.47). Leg formula 1423.

***Epigyne*** (Fig. 5C, D): epigyne with a rugby-ball-shaped atrium, laterally with triangular pockets. Copulatory openings situated in anterior part of atrium. Copulatory ducts coiled once. Spermathecae spherical. Fertilization ducts arising from posterior part of spermathecae.

***Colouration in ethanol*** (Fig. 7D, H, L): carapace rounded and pale yellow, medially with a wide red line. Sternum, chelicerae, labium, palps, and legs pale yellow. Opisthosoma pale yellow, posteriorly extending with black, flattened spines; dorsum with white patches and reddish-brown transverse lines; venter laterally with black transverse line.

**Male**: unknown.

##### Distribution.

Known only from the type locality (China: Hainan).

#### ﻿Genus Physcoa

Taxon classificationAnimaliaAraneaeTheridiidae

Thorell, 1895 stat. rev.

F016F22D-E27E-5EE0-B98C-22998C8C0937

Physcoa : Thorell 1895: 82.

##### Type species.

*Physcoa scintillans* Thorell, 1895, comb. rev. from Myanmar.

##### Diagnosis.

Members of the genus *Physcoa* Thorell, 1895, stat. rev. can be distinguished from the true *Chrysso* (cf. Figs 8–12; fig. 34A–E in Zhu 1998; fig. 111.1–3 in Song et al. 2006; figs 481–492 in Sen et al. 2015 and figs 1–7, 9, 11, 13, 15, 21–31, 34, 35 in Levi 1957; figs 7, 8, 12 in Buckup and Marques 1996) by the following combination of characteristics: (1) cymbium with a prolateral distal projection (except *P. cyclocera* (Zhu, 1998), comb. nov.) (vs cymbium without projection); (2) embolus C-shaped, with embolic projection in some species (vs filiform and complexly elongating, without embolic projection); and (3) copulatory ducts spherical, smaller than spermathecae (except *P. cyclocera* (Zhu, 1998), comb. nov.) (vs sac-like, much larger than spermathecae).

**Figure 9.**
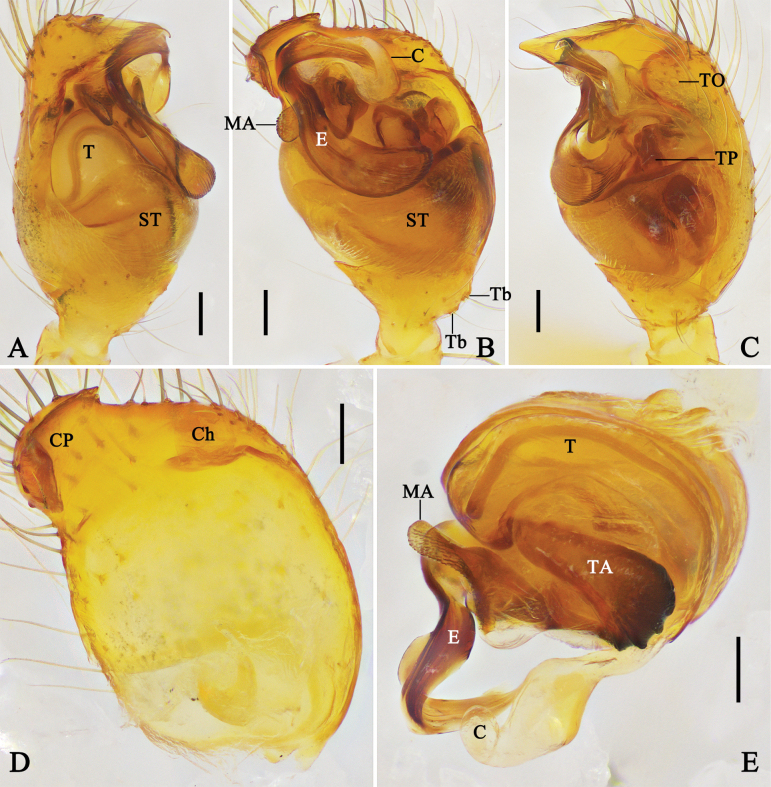
Left male palp of *Physcoa scintillans* Thorell, 1895, comb. rev. **A.** Prolateral view; **B.** Ventral view; **C.** Retrolateral view; **D.** Cymbium, ventral view; **E.** Expanded bulb, apical view. Abbreviations: C = conductor; Ch = cymbial hood; CP = cymbial projection; E = embolus; MA = median apophysis; ST = subtegulum; T = tegulum; TA = tegular apophysis; Tb = trichobothria; TO = tarsal organ; TP = tegular pit. Scale bars: 0.1 mm.

Members of the genus *Physcoa* Thorell, 1895, stat. rev. is similar to *Argyrodes* Simon, 1864 (cf. Figs 8, 9, 11, 12 and fig. 52 in Agnarsson et al. 2007; figs 2–8 in Kaya et al. 2010) in having a posterior extension on dorsal opisthosoma and the presence of prolateral cymbial projection, but it can be distinguished by: (1) bulb-cymbium-lock mechanism hood-like (vs hook-like); (2) tegular apophysis short, not visible before expanded, covered by bulb (vs long, protrusive, and visible before expanded); (3) embolus C-shaped (vs triangular); and (4) copulatory ducts spherical (vs tubular).

##### Description.

***Habitus*** (Figs 13, 14): male total length 2.01–3.92, female 2.48–7.70. Carapace rounded or oval. Sternum triangular. Chelicerae with two or three promarginal teeth, without retromarginal teeth. Leg formula 1423. Retrolateral part of patella of legs with apophysis. Dorsal part of opisthosoma laterally with two humps and posterior part with extension beyond spinnerets. Colulus or colular setae absent.

***Male palp*** (Figs 8, 9, 11, 12): tibia wider than long, with distal part almost 2–3 times wider than basal part in ventral view; retrolateral part with one or two trichobothria. Cymbium almost as long as wide or slightly longer than wide, prolateral-distal part with a projection; cymbial hood located at retrolateral-distal margin of cymbium, wider than long; tarsal organ enlarged, three times larger than setal sockets. Subtegulum bowl-like. Tegulum retrolaterally with a rounded pit. Median apophysis visible and tegular apophysis not visible in ventral view; tegular apophysis almost as large as median apophysis. Conductor incrassate, membranous, supporting embolic tip. Embolus wide, C-shaped, basal hook spherical, forming a lock mechanism with tegular pit.

**Figure 10.**
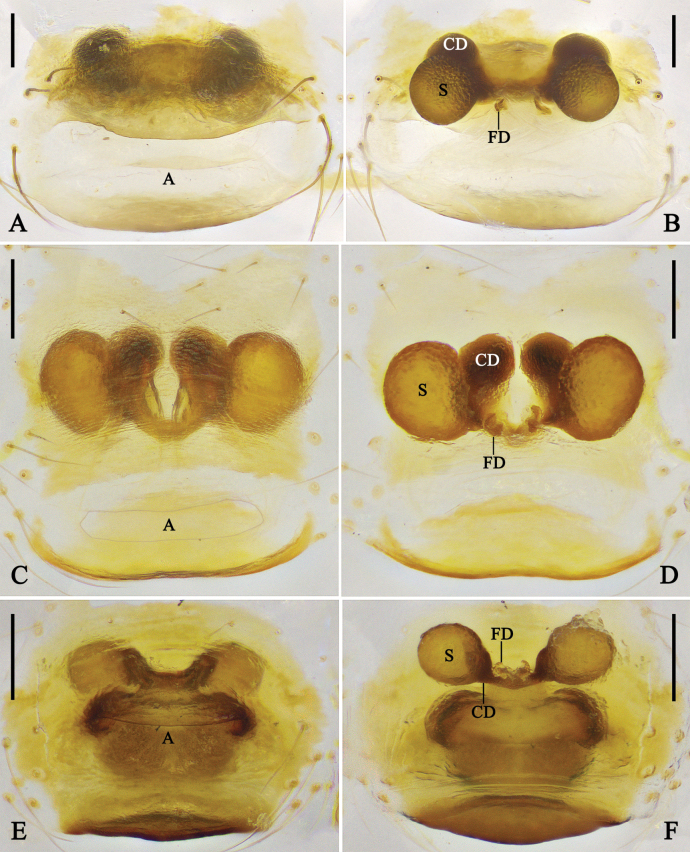
Female genitalia of *Physcoa* spp. **A, B.***P. scintillans* Thorell, 1895, comb. rev.; **C, D.***P. trimaculata* (Zhu, Zhang & Xu, 1991), comb. nov.; **E, F.***P. trispinula* (Zhu, 1998), comb. nov.; **A, C, E.** Epigyne, ventral view; **B, D, F.** Vulva, dorsal view. Abbreviations: A = atrium; CD = copulatory duct; FD = fertilization duct; S = spermatheca. Scale bars: 0.1 mm.

***Epigyne*** (Fig. 10): epigyne with a short atrium, wider than 2/3 the width of epigynal field. Copulatory ducts almost spherical. Spermathecae spherical, wider than copulatory ducts. Fertilization ducts originating from posterior part of spermathecae.

**Figure 11.**
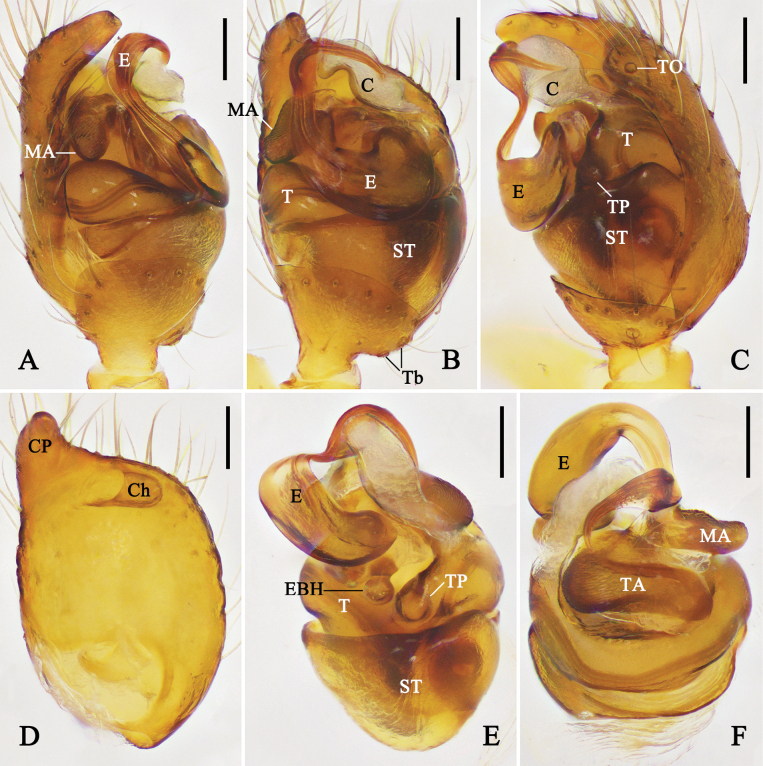
Left male palp of *Physcoa trimaculata* (Zhu, Zhang & Xu, 1991), comb. nov. **A.** Prolateral view; **B.** Ventral view; **C.** Retrolateral view; **D.** Cymbium, ventral view; **E.** Expanded bulb, retrolateral view; **F.** Same, dorsal view. Abbreviations: C = conductor; Ch = cymbial hood; CP = cymbial projection; E = embolus; EBH = embolic basal hook; MA = median apophysis; ST = subtegulum; T = tegulum; TA = tegular apophysis; Tb = trichobothria; TO = tarsal organ; TP = tegular pit. Scale bars: 0.1 mm.

**Figure 12.**
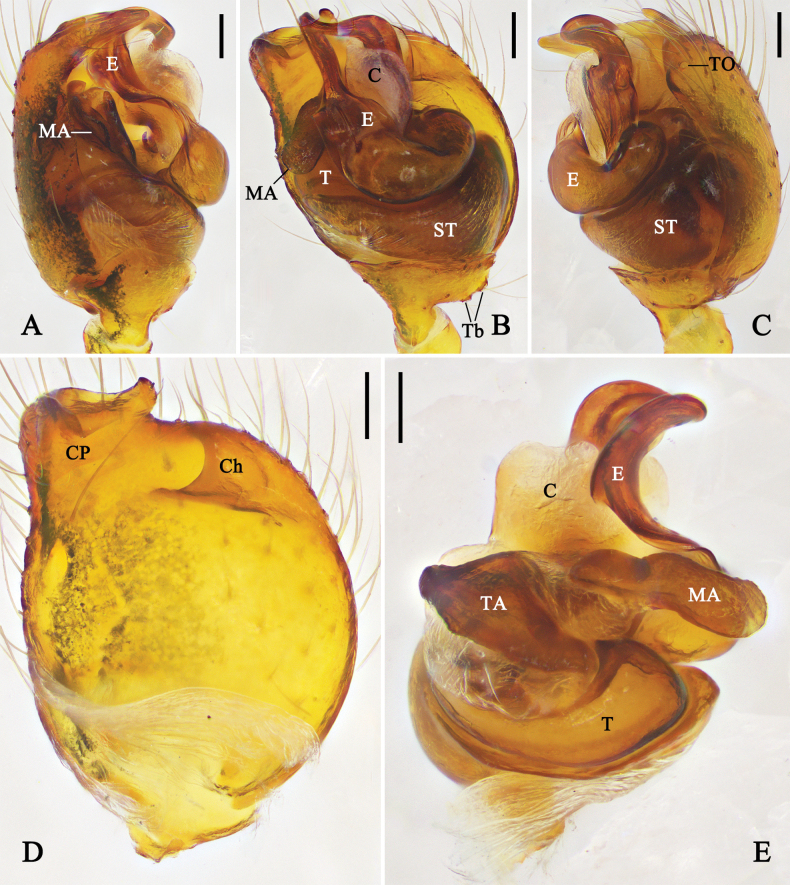
Left male palp of *Physcoa wenxianensis* (Zhu, 1998), comb. nov. **A.** Prolateral view; **B.** Ventral view; **C.** Retrolateral view; **D.** Cymbium, ventral view; **E.** Expanded bulb, dorsal view. Abbreviations: C = conductor; Ch = cymbial hood; CP = cymbial projection; E = embolus; MA = median apophysis; ST = subtegulum; T = tegulum; TA = tegular apophysis; Tb = trichobothria; TO = tarsal organ. Scale bars: 0.1 mm.

**Figure 13.**
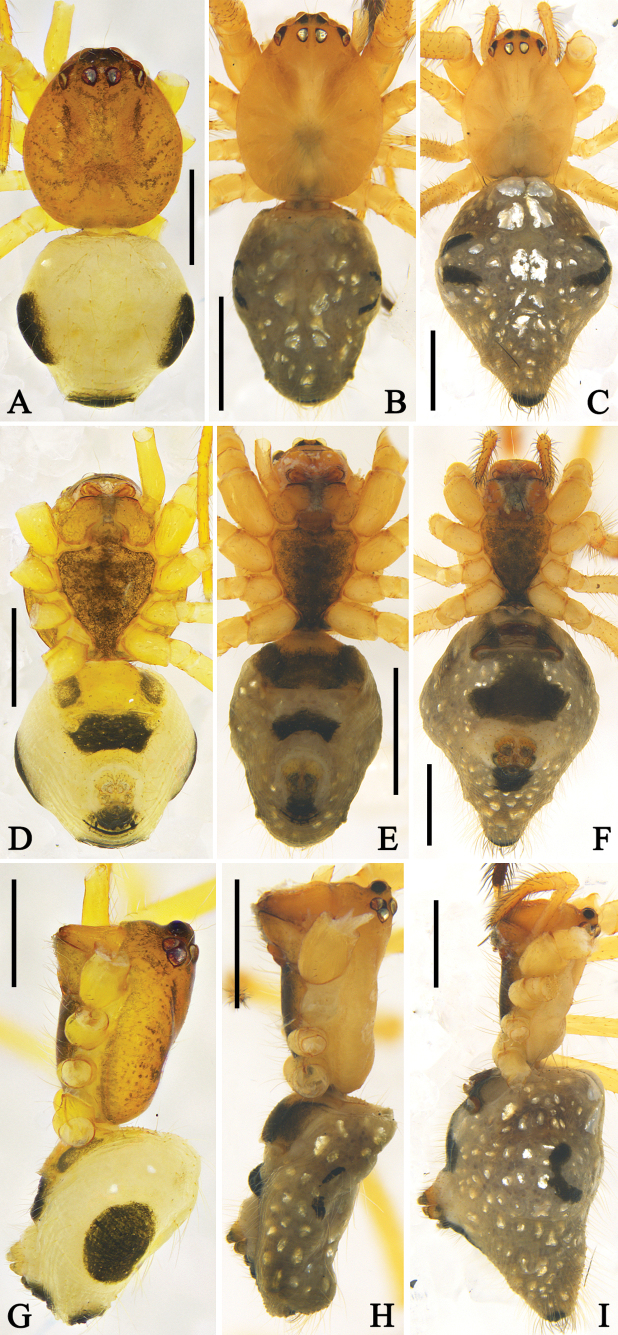
Habitus of *Physcoa* spp. **A, D, G.** Male of *P. oxycera* (Zhu & Song, 1993), comb. nov.; **B, E, H.** Male of *P. scintillans* Thorell, 1895, comb. rev.; **C, F, I.** Female of *P. scintillans* Thorell, 1895, comb. rev.; **A–C.** Dorsal view; **D–F.** Ventral view; **G–I.** Lateral view. Scale bars: 0.5 mm (**A, D, G**); 1 mm (**B, C, E, F, H, J**).

**Figure 14.**
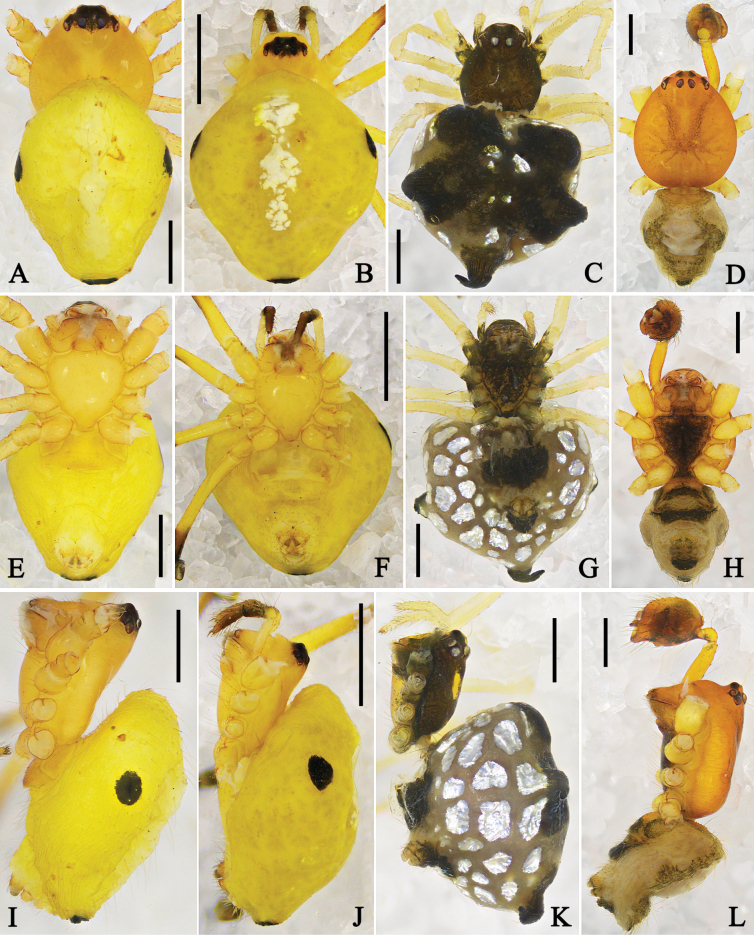
Habitus of *Physcoa* spp. **A, E, I.** Male of *P. trimaculata* (Zhu, Zhang & Xu, 1991), comb. nov.; **B, F, J.** Female of *P. trimaculata* (Zhu, Zhang & Xu, 1991), comb. nov.; **C, G, K.** Female of *P. trispinula* (Zhu, 1998), comb. nov.; **D, H, L.** Male of *P. wenxianensis* (Zhu, 1998), comb. nov.; **A–D.** Dorsal view; **E–H.** Ventral view; **I–L.** Lateral view. Scale bars: 0.5 mm (**A, C–E, G–I, K, L**); 1 mm (**B, F, J**).

##### Composition.

*Physcoa angula* (Tikader, 1970), comb. nov., *P. cyclocera* (Zhu, 1998), comb. nov., *P. fanjingshan* (Song, Zhang & Zhu, 2006), comb. nov., *P. oxycera* (Zhu & Song, 1993), comb. nov., *P. scintillans* Thorell, 1895, comb. rev., *P. trimaculata* (Zhu, Zhang & Xu, 1991), comb. nov., *P. trispinula* (Zhu, 1998), comb. nov., and *P. wenxianensis* (Zhu, 1998), comb. nov.

##### Remarks.

Although the specimens of *Chrysso angula*, *Chr. cyclocera*, and *Chr. fanjingshan* were unavailable for examination, the illustrations and descriptions of *Chr. angula* and *Chr. fanjingshan* (fig. 111.1–3 in Song et al. 2006; figs 481–492 in Sen et al. 2015) clearly show the following characteristics of *Physcoa*: a projection on prolateral distal cymbium, the C-shaped embolus (male of *Chr. fanjingshan* unknown), a wide and short epigynal atrium, and the spherical copulatory duct. Despite *Chr. cyclocera* (fig. 34A–E in Zhu 1998) lacking a cymbial projection, this species exhibits a C-shaped embolus with an embolic projection, which is similar to *P. oxycera* comb. nov. Therefore, we transfer these three species to *Physcoa*.

##### Distribution.

China, India, Japan, Korea, Myanmar, Philippines, Thailand.

#### ﻿ Physcoa oxycera

Taxon classificationAnimaliaAraneaeTheridiidae

(Zhu & Song, 1993) comb. nov.

A3F677AD-C8FB-59D8-B9A7-D5E147AFCD60

Figs 8, 13A, D, G

Chrysso oxycera : Song et al. 1993: 857, fig. 10A–D (male); Zhu 1998: 63, fig. 35A–D (male); Song et al. 1999: 103, fig. 50H, I (male).

##### Material examined.

1 male (QZMS04208): China – Hubei Province • Enshi Tujia and Miao Autonomous Prefecture, Xuan’en County, Qizimeishan National Nature Reserve, Shadaogou Town, Baishuihe Village; 29.9238°N, 109.7360°E; elev. 843 m; 23–24 July 2023; C.H. Hu and M. Wei leg.

##### Diagnosis.

*Physcoa oxycera* (Zhu & Song, 1993), comb. nov. is similar to *P. cyclocera* (Zhu, 1998), comb. nov. (cf. Fig. 8 and fig. 34D, E in Zhu 1998) in having an embolic projection, but can be distinguished from *P. cyclocera* by: (1) retrolateral tibia with one trichobothrium (vs with two trichobothria); (2) prolateral distal cymbium with a projection (vs cymbium without projection); and (3) embolic base with a tiny, tooth-like projection (vs without basal projection).

##### Description.

See Zhu (1998).

##### Remarks.

This species is transferred to *Physcoa* based on its similarity to the type species: i.e. prolateral distal cymbium with projection and cymbial hood located on retrolateral distal cymbium, and the embolus C-shaped.

##### Distribution.

China (Hubei, new provincial record; Fujian).

#### ﻿ Physcoa scintillans

Taxon classificationAnimaliaAraneaeTheridiidae

Thorell, 1895 comb. rev.

D071A211-79E1-57C5-9CA1-DBE53E70B6AF

Figs 9, 10A, B, 13B, C, E, F, H, I

Physcoa scintillans : Thorell 1895: 83 (female).
Argyria venusta : Yaginuma 1957: 11, fig. 1A–J (male, female).
Argyroaster venusta : Yaginuma 1958: 37; Yaginuma 1960: 38, fig. 36 (male).
Chrysso venusta : Levi 1962: 209, figs 3–5 (male, female); Zhu 1998: 59, fig. 32A–D (male, female, synonym of Chr. bidens).
Chrysso bidens : Xing et al. 1994: 164, figs 1–5 (male, female).
Chrysso scintillans : Yoshida 2001: 174, figs 66–71 (male, female, transfer from Physcoa, synonym of Chr. venusta); Breitling 2015: 2 (synonym of Linyphia bilobata); Kim, 2021: 40, fig. 13A–J, pl. 7 (male, female). Naya 2024: 61, f. 1–4 (male, female).
Linyphia bilobata : Roy et al. 2015: 62, figs 1–7 (female).^1^

##### Material examined.

9 males, 14 females (QZMS00606, 00765, 00993, 02261–02264, 02269, 02396, 02685–02689, 03543, 04207, 04469–04475): China – Hubei Province • Enshi Tujia and Miao Autonomous Prefecture, Xuan’en County, Qizimeishan National Nature Reserve; 29.7400°N–30.0764°N, 109.5919°E–109.7763°E; elev. 585–1822 m; 30 June–31 July 2023; C.H. Hu and M. Wei leg.

##### Diagnosis.

*Physcoa scintillans* Thorell, 1895, comb. rev. is similar to *P. trimaculata* (Zhu, Zhang & Xu, 1991), comb. nov. (cf. Figs 9, 10A, B and Figs 10C, D, 11) in having digitiform median apophysis in ventral view, similar embolic shape, epigynal atrium posteriorly situated, but can be distinguished from *P. trimaculata* by: (1) prolateral part of tegulum almost as long as wide (vs wider than long); (2) embolic tip almost as wide as median apophysis (vs thinner than median apophysis); (3) atrium as wide as and half the length of epigynal field (vs almost 3/5 the width and 1/8 the length of epigynal field); and (4) copulatory duct situated at anterior part of spermatheca (vs situated between spermathecae).

##### Description.

See Kim (2021).

##### Distribution.

China (Anhui; Fujian; Guizhou; Hainan; Hubei; Hunan; Sichuan; Taiwan; Yunnan; Zhejiang), India, Japan, Korea, Myanmar, Philippines.

#### ﻿ Physcoa trimaculata

Taxon classificationAnimaliaAraneaeTheridiidae

(Zhu, Zhang & Xu, 1991) comb. nov.

5CE8BBC6-FD8F-5AF6-9A65-521C7FFCA797

Figs 10C, D, 11, 14A, B, E, F, I, J

Chrysso trimaculata : Zhu et al. 1991: 174, figs 8–12 (male, female); Yoshida 1993: 31, figs 13, 14 (male); Zhu 1998: 58, fig. 31A–F (male, female); Song et al. 1999: 107, fig. 50F, G, O, P (male, female); Yin et al. 2012: 302, fig. 113a–e (male, female); Chotwong and Tanikawa 2013: 1, figs 1, 2, 7, 8 (male, female); Lin et al. 2023: 520 (synonym of Chr. hejunhuai).
Chrysso hejunhuai : Barrion et al. 2013: 35, fig. 39A–H (male).

##### Material examined.

2 males, 2 females (LJ201800703, 201800708, 201800769, 201800770): China – Hainan Province • Wuzhishan City, Shuiman Town, Wuzhishan; 18.8825°N, 109.6609°E; elev. 140 m; 15 April 2018; F.X. Liu and Z.C. Li leg. 1 female (HB-IV-180108): Hubei Province • Enshi Tujia and Miao Autonomous Prefecture, Xuan’en County, Changtanhe Dong Autonomous Town; 30.0322°N, 109.6517°E; elev. 725 m; 11 August 2018; J. Chen et al. leg. 1 female (LJ201904331): Yunnan Province • Xishuangbanna, Mengla County, Wangtianshu; 21.6250°N, 101.5872°E; elev. 710 m; 25 November 2019; J. Liu leg.

##### Diagnosis.

See above diagnosis under *Physcoa scintillans* Thorell, 1895, comb. rev.

##### Description.

See Chotwong and Tanikawa (2013).

##### Remarks.

This species is transferred to *Physcoa* based on its similarity to the type species: i.e. prolateral distal cymbium with a projection, cymbial hood located on retrolateral distal part of cymbium, embolus C-shaped, epigyne with a wide and short atrium, and the copulatory ducts spherical and narrower than spermathecae.

This species is probably a junior synonym of *P. angula* (Tikader, 1970), comb. nov. from India, based on similarities in somatic characters and copulatory organ morphology (three black spots on the opisthosoma, copulatory ducts located between the spermathecae fig. 8a, b in Tikader 1970; figs 481–488 in Sen et al. 2015; figs 1–11 in Sekhar and Sunil Jose 2020). However, *P. angula* lacks clear photos, and the types are unavailable for examination. Further research is needed to resolve this issue conclusively.

##### Distribution.

China (Hubei, new provincial record; Yunnan, new provincial record; Fujian; Hainan; Hunan; Guizhou; Taiwan), Thailand.

#### ﻿ Physcoa trispinula

Taxon classificationAnimaliaAraneaeTheridiidae

(Zhu, 1998) comb. nov.

DFAA836C-A5A6-5CF8-A344-65D0867ADA03

Figs 10E, F, 14C, G, K

Chrysso trispinula : Zhu 1998: 50, fig. 24A–D (female); Song et al. 1999: 107, fig. 51A, B (female); Yin et al. 2012: 304, fig. 114a–c (female); Jin 2018: 19, fig. 4–9A–D, pl. 4 (female).

##### Material examined.

1 female (HB-IV-180108): China – Hubei Province • Enshi Tujia and Miao Autonomous Prefecture, Xuan’en County, Changtanhe Dong Autonomous Town; 30.0322°N, 109.6517°E; elev. 725 m; 11 August 2018; J. Chen et al. leg.

##### Diagnosis.

The female of *Physcoa trispinula* (Zhu, 1998), comb. nov. can be distinguished from all congeners by the posterior margin of epigyne with a labiate sclerotized part.

##### Description.

See Zhu (1998).

##### Remarks.

This species is transferred to *Physcoa* based on its similarity to the type species: i.e. epigyne with a wide and short atrium, copulatory ducts spherical and narrower than spermathecae.

##### Distribution.

China (Hubei, new provincial record; Hainan; Hunan; Zhejiang).

#### ﻿ Physcoa wenxianensis

Taxon classificationAnimaliaAraneaeTheridiidae

(Zhu, 1998) comb. nov.

7E0E57BD-F8C1-5E56-896E-FC4C70C68FCC

Figs 12, 14D, H, L

Chrysso wenxianensis : Zhu 1998: 64, fig. 36A–D (male); Song et al. 1999: 107, fig. 51N, O (male); Yin et al. 2012: 308, fig. 117a–c (male).

##### Material examined.

3 males (LJ201801903, 201801951, HN-IV-180001): China – Hunan Province • Zhangjiajie City, Sangzhi County, Tianpingshan Nature Reserve, Tianpingshan Forest Farm, Gongtongyuan; 29.7833°N, 110.0869°E; elev. 1401 m; 1 June 2018; R. Zhong et al. leg.

##### Diagnosis.

The male of *Physcoa wenxianensis* (Zhu, 1998), comb. nov. can be distinguished from all congeners by: (1) retrolateral part of cymbial projection with a digitiform projection; (2) median apophysis rolled in prolateral view; and (3) embolic tip covered by conductor in ventral view.

##### Description.

See Zhu (1998).

##### Remarks.

This species is transferred to *Physcoa* based on its similarity to the type species: i.e. prolateral distal cymbium with a projection, cymbial hood located on retrolateral distal part of cymbium, and the embolus C-shaped.

##### Distribution.

China (Gansu; Hunan).

## Supplementary Material

XML Treatment for Chrysso

XML Treatment for Megama

XML Treatment for Megama decimaculata

XML Treatment for Megama bimaculata

XML Treatment for Meotipa

XML Treatment for Meotipa cordata

XML Treatment for Meotipa lingchuanensis

XML Treatment for Meotipa vittiforma

XML Treatment for Physcoa

XML Treatment for Physcoa oxycera

XML Treatment for Physcoa scintillans

XML Treatment for Physcoa trimaculata

XML Treatment for Physcoa trispinula

XML Treatment for Physcoa wenxianensis

## Footnotes

All references: see WSC (2025).

## ﻿Additional information

### Use of AI

No use of AI was reported.

### Author ORCIDs

Changhao Hu https://orcid.org/0009-0007-5591-3121

Rui Zhong https://orcid.org/0000-0001-5910-7302

Jie Liu https://orcid.org/0000-0001-7744-9744

Zichang Li https://orcid.org/0000-0002-0357-0864

### Data availability

All of the data that support the findings of this study are available in the main text.
